# Viral Replication and Lung Lesions in BALB/c Mice Experimentally Inoculated with Avian Metapneumovirus Subgroup C Isolated from Chickens

**DOI:** 10.1371/journal.pone.0092136

**Published:** 2014-03-17

**Authors:** Li Wei, Shanshan Zhu, Ruiping She, Fengjiao Hu, Jing Wang, Xu Yan, Chunyan Zhang, Shuhang Liu, Rong Quan, Zixuan Li, Fang Du, Ting Wei, Jue Liu

**Affiliations:** 1 Beijing Key Laboratory for Prevention and Control of Infectious Diseases in Livestock and Poultry, Institute of Animal Husbandry and Veterinary Medicine, Beijing Academy of Agriculture and Forestry Sciences, Beijing, People’s Republic of China; 2 College of Veterinary Medicine, China Agricultural University, Beijing, People’s Republic of China; 3 Molecular Virology Laboratory, QIMR Berghofer Medical Research Institute, Queensland, Australia; French National Centre for Scientific Research, France

## Abstract

Avian metapneumovirus (aMPV) emerged as an important respiratory pathogen causing acute respiratory tract infection in avian species. Here we used a chicken aMPV subgroup C (aMPV/C) isolate to inoculate experimentally BALB/c mice and found that the aMPV/C can efficiently replicate and persist in the lungs of mice for at least 21 days with a peak viral load at day 6 postinoculation. Lung pathological changes were characterized by increased inflammatory cells. Immunochemical assay showed the presence of viral antigens in the lungs and significant upregulation of pulmonary inflammatory cytokines and chemokines including MCP-1, MIP-1α, RANTES, IL-1β, IFN-γ, and TNF-α were detected following inoculation. These results indicate for the first time that chicken aMPV/C may replicate in the lung of mice. Whether aMPV/C has potential as zoonotic pathogen, further investigation will be required.

## Introduction

Avian metapneumovirus (aMPV), belonging to the *Metapneumovirus* genus within the family *Paramyxoviridae*, causes an acute respiratory disease characterized by nasal and ocular discharge, foamy conjunctivitis, facial congestion and swollen infraorbital sinuses, as well as egg drops and poor egg quality in turkeys, chickens and ducks [1]. aMPV is an enveloped virus containing a single stranded, negative sense RNA genome with a total length of approximately 13 kb, which organized in the order 3′-leader-N-P-M-F-M2-SH-G-L-trailer-5′ [2]. Four subgroups (A, B, C, and D) of aMPV have been identified based upon genetic and antigenic properties of viral attachment (G) glycoprotein. After its first description in South Africa during 1978 [3], aMPV subgroups A and B, were mainly found thereafter in Europe, Asia and many other parts of the world, in turkeys and/or chickens. Subgroup C aMPV (aMPV/C) was first reported in turkeys in the USA in 1996 [4] and subsequently isolated from farmed ducks [5] and pheasants [6], some wild birds [7] and our most recent report from chickens [20]. Analysis of aMPV/C isolates revealed low sequence identity to subgroups A and B, exhibiting no or limited cross-reactivity with aMPV subgroups A and B in virus neutralization and enzyme-linked immunosorbent assay. Research data [8], [9], [10] further indicated that aMPV/C had closer genetic and antigenic relatedness to human metapneumovirus (hMPV), within the same genus *Metapneumovirus* as aMPV, than to other aMPV subgroups, thereby emphasizing the need for a better understanding of aMPV/C pathogenesis.

hMPV causes severe respiratory diseases including bronchiolitis/bronchitis and pneumonia occurring in young children, elderly individuals, as well as immunocompromised persons [11]. It was reported that hMPV can infect and replicate in several small-animal and nonhuman primate models [12], [13], [14]. BALB/c mouse, a good and convenient experimental animal model, has been used to study the pathogenesis and immunity of hMPV infection [15], [16], [17], [18], [19]. hMPV replication has been found in the lung of experimentally infected BALB/c mice and was associated with transient weight loss [15]. Recently, we for the first time isolated and characterized an aMPV/C strain JC from Chinese local meat-type commercial chickens with severe respiratory signs [20]. Sequence analysis showed that the chicken aMPV/C strain JC is more closely related to hMPV strain BJ1816 (78.5%) isolated in China than to other aMPV/C isolates (75.5–77.8%) as compared to matrix gene sequences. It has been reported previously that hMPV could cause clinical signs such as nasal discharge in the inoculated turkey [21]. Thus, this prompts us to investigate whether chicken aMPV/C strain JC which shows closer homology to hMPV can infect and replicate in the respiratory tract of experimentally inoculated mammal animals.

In the present study, we investigated the pathogenesis of the chicken aMPV/C inoculation in the lung of BALB/c mice. Our results indicate that BALB/c mice efficiently support aMPV/C replication, with significant lung inflammation, fever, and being depressed, which showed similar infectivity as observed for hMPV in BALB/c mice. This study demonstrates for the first time that chicken aMPV/C can infect BALB/c mice and persist as infectious virus in the lungs of inoculated mice for several weeks.

## Materials and Methods

### Ethics statement

This study was conducted according to the animal welfare guidelines of the World Organization for Animal Health [22], and approved by the Animal Care and Use Committee of Institute of Animal Husbandry and Veterinary Medicine Beijing Academy of Agriculture and Forestry Sciences.

### Virus and cells

aMPV subgroup C (aMPV/C) strain JC isolated from Chinese local meat-type chickens with respiratory syndrome as described recently [20] was used for this study. The stock virus was passaged twelve times in monkey Vero cells before one freeze-thaw cycle and clarification to release infectious virus. The presence of chicken aMPV/C isolate JC was confirmed by reverse transcriptase PCR (RT-PCR), immunofluorescence assay, and detection of cytopathic changes in cells (cell rounding and syncytial formation). The virus was titrated by serial dilutions onto Vero cells and found to be 10^4.25^ 50% tissue culture infectious dose (TCID_50_) per 0.1 milliliter.

### BALB/c mice

A total of 120 8-week-old, specific-pathogen–free female BALB/c mice were purchased from Vital River Laboratories, Beijing, China. The mice were randomly allocated two groups and housed in isolation rooms in filter-top cages and fed sterilized food and water *ad libitum*. One group (60 of mice) was inoculated intranasally and intraperitoneally with the aMPV/C strain JC at 10^4.25^ TCID_50_ in a total volume of 100 μl that harvested from Vero cell, whereas the other group was set as negative control and sham inoculated with 100 μl of Vero cell supernatant. The animals were monitored daily for mortality, weight loss, and presence of any respiratory symptoms. Rectal temperature for the two groups of mice was measured. The mean temperature of sham-inoculated mice at the indicated times was considered the reference for interpretation of the results. At serial times postinoculation (days 1, 2, 3, 4, 5, 6, 7, 10, 14, and 21), the lungs and blood samples were collected from 6 mice from both aMPV/C- and sham-inoculated groups.

### Virus titration in lungs

For virus titer assay, the lungs of the all experimental mice were removed and quickly frozen in –80°C freezer. Lung tissues were homogenized in 1 ml of Dulbecco’s modified Eagle’s medium (DMEM) and centrifuged at 10,000 × g for 1 min at 4°C, and the supernatants were laid on Vero monolayers for virus titration. Virus titers were determined as described elsewhere [23] and expressed as TCID_50_ per lung, corresponding to ∼0.05 g.

### Quantitative reverse transcription (RT)-PCR analysis

The levels of viral RNA in the lungs were assessed by quantitative RT-PCR (qRT-PCR) at the indicated time points after aMPV/C inoculation. Total RNAs were isolated from lung tissue of inoculated- or sham-inoculated mice by using RNeasy Mini kit (Qiagen) for qRT-PCR. The sense primer (5′- GTCAATTCAGCCAAGGCAGT-3′) and the antisense primer (5′- GGGGCAATCCTAGCTTGAGT-3′) designed for this study were used to amplify a 200-bp nucleotide region of M gene. qRT-PCR protocol followed the instructions of an iScript™ One-Step RT-PCR Kit with SYBR® Green (Bio-Rad). The qRT-PCR parameters consisted of a cDNA synthesis at 50°C for 10 min and reverse transcriptase inactivation at 95°C for 10 min, and then 35 cycles of denaturation at 95°C for 10 s and annealing at 55°C for 30 s. For a standard curve, serial dilutions of plasmid pMD18-M (M gene cloned into pMD-18) were used to quantify the virus genomic copy number. Each assay was run in triplicate.

### Pathological examination

The mice were sacrificed and lung samples were collected at the indicated time points after aMPV/C inoculation, and fixed by immersion in 2.5% glutaraldehyde-polyoxymethylene solution. Fixed tissues were dehydrated, embedded in paraffin wax, sectioned at 4 μm thickness, and then stained with hematoxylin and eosin (HE) for light microscopic observation. For lung change evaluation, four types of histopathological changes were scored independently (peribronchiolitis, perivasculitis, interstitial pneumonitis, and alveolitis) based on a scale of 0 (no change) to 4 (Maximum change) as observed for hMPV infection of mice [15], [24], [25].

### Immunohistochemical staining

The lung tissues for histology were subjected to immunohistochemical analysis for detecting the presence of aMPV viral antigen. Briefly, the deparaffinized sections were incubated with a rabbit polyclonal antibody raised against a polypeptide located in N protein of all aMPV subgroups [26], followed by incubation with biotinylated secondary antibody and horseradish peroxidase-labeled avidin-biotin chain working fluid (Beijing Zhong Shan Golden Bridge Biotechnology Co., Ltd., China) before the addition of 3,3′-diaminobenzidine (Zymed Laboratories Inc., San Diego, CA) as a substrate. The tissue sections were then counterstained with hematoxylin, dehydrated, and mounted with neutral gums. Sections from the sham-inoculated mice served as negative controls. The results were expressed as an area density of positive signals, which was obtained by dividing the area of positive signals by the area of the whole field under the microscope at 400 × magnification.

### Neutralization assay

aMPV/C neutralizing antibody titers were determined by using an end-point dilution reduction assay. Briefly, polled serum (*n*  =  6) collected from each time point after aMPV/C inoculation was diluted twofold in serum-free DMEM and mixed 1:1 (vol/vol) with 200 TCID_50_ of aMPV/C strain JC. After incubation 1 h at 37°C, the reaction mixtures were added to 95% confluent Vero cells in 96-well plates. Each dilution was inoculated into four wells. At 168 h post-inoculation, the cell monolayers were monitored for cytopathic effects. Neutralizing antibody end-point titers were calculated by the method of Reed and Muench method and reported as the reciprocal value of the highest serum dilution that neutralized 200 50% TCID_50_ of aMPV/C.

### Pulmonary cytokine levels

Cytokine levels, including monocyte chemotactic protein 1 (MCP-1), macrophage inflammatory protein 1α (MIP-1α), RANTES, IL-1β, interferon (IFN)-α, IFN-γ, tumor necrosis factor (TNF)-α were measured by respective mouse enzyme-linked immunosorbent assay (ELISA) kit obtained from Invitrogen according to the protocols of the manufacturer. Briefly, lung tissues (*n* = 6 for each) at the indicated time points after aMPV/C inoculation were homogenized (25% w/v) in phosphate-buffered saline (PBS) followed by dilution with Standard Diluent Buffer. Samples were centrifuged at 13,500 × g for 10 min at 4°C, and 100 μl of the supernatant was used for cytokine quantification by ELISA.

### Statistical analysis

Results are presented as averages ± the standard deviation or standard errors of the means, as indicated. Statistical comparisons are made by using Student’s *t* test, and differences between groups were considered significant if the *P* value was < 0.05.

## Results

### Clinical manifestations of aMPV/C inoculation in mice

**Figure 1:**
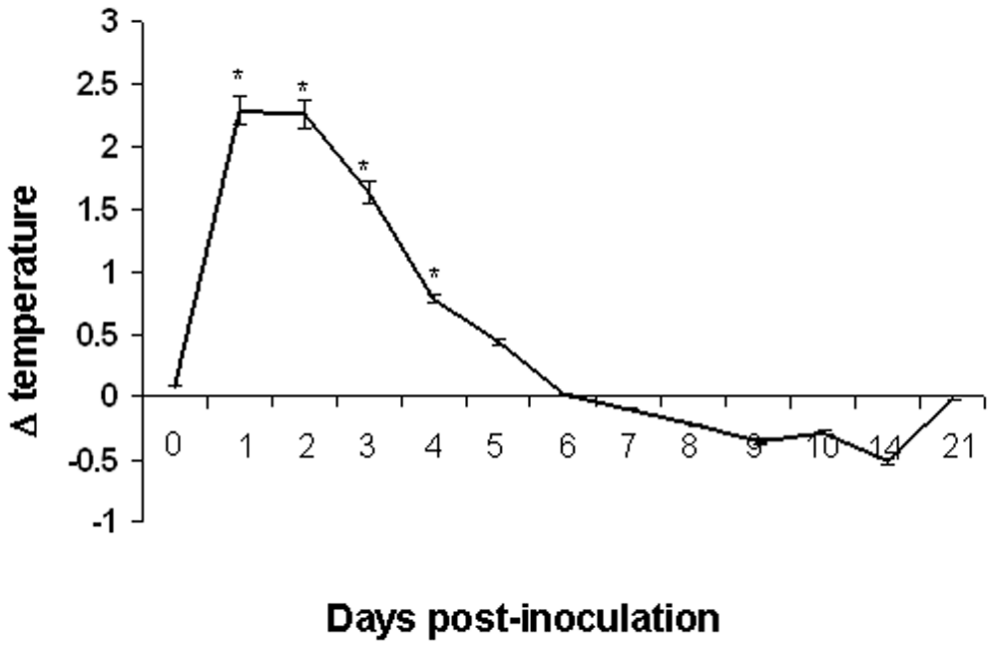
Temperature kinetics in aMPV/C-inoculated BALB/c mice. The temperature of sham-inoculated mice was used as a reference to reduce biases due to circadian rhythms. Significant temperature change is expressed as *p* < 0.05 (*) for a comparison of aMPV/C-inoculated and sham-inoculated mice at the indicated time points after inoculation. Data are shown as the mean ± standard deviations.

BALB/c mice were inoculated with 10^4.25^ TCID_50_ of chicken aMPV/C strain JC and were observed daily. A cohort was observed for signs of disease such as being ataxia, ruffled fur, tendency to huddle, or being less active, inactive from day 1 through day 6 followed by recovery in the aMPV/C-inoculated mice. As shown in Fig. 1, the chicken aMPV/C-inoculated mice exhibited fever at day 1 through day 5 postinoculation, the rectal temperature of the inoculated mice increased dramatically to reach a value as high as +2.29±0.13°C (*p*<0.05) at day 1 thereafter declined as compared to the sham-inoculated mice. The temperature returned to normal levels at day 6 postinoculation. In addition, the virus-inoculated mice showed obvious decreased rectal temperature at day 14 postinoculation. None of the aMPV/C-inoculated mice lost weight or died throughout the study. None of the sham-inoculated mice showed any signs of illness or weight loss.

### Virus amounts in lungs

**Figure 2:**
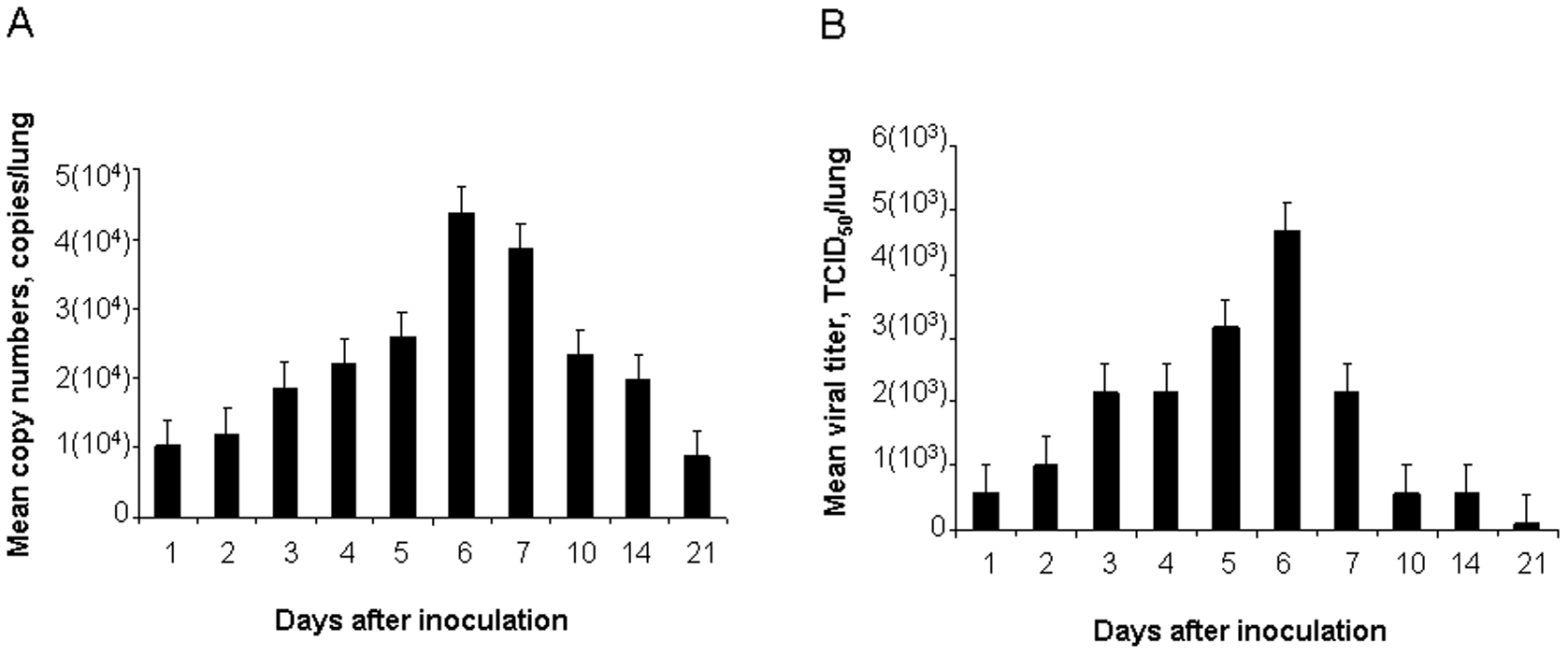
aMPV/C replication in the lungs of inoculated mice (*n*  =  6). Lung homogenates from various times postinoculation were subject to determine aMPV copy numbers using a qRT-PCR (A) or were serially diluted and incubated with Vero cells for viral titration (B).

The levels of viral RNA and virus titers in the lungs of mice were assessed at 1, 2, 3, 4, 5, 6, 7, 10, 14, and 21 days after aMPV/C inoculation. qRT-PCR analysis indicated that inoculation of aMPV/C led to progressive accumulation of viral RNA in the lungs of mice at day 6 postinoculation, with the maximal copy numbers being 4.83×10^4^ copies/lung, which decreased thereafter (Fig. 2A). Later on, viral copy numbers did not vary significantly in positive samples and were between 2.34×10^4^ and 0.87×10^4^ copies/lung from days 7 to 21. Consistent with the results shown in Fig. 2A, there was a time-dependent increase in the virus titers in the aMPV/C-inoculated mice at 6 days postinoculation which declined thereafter. At 6 days after inoculation, virus titer reached the highest level (10^3.67^ TCID_50_/lung) and then dropped down. Virus loads can be detected in the aMPV/C-inoculated mice throughout the experiment (21 days). This showed that the virus shedding in the inoculated mice last at least 21 days. Negative-control mice were negative for aMPV subgroup C virus or viral RNA throughout the study (data not shown). In the present study, the aMPV/C-inoculated mice exhibited fever and other clinical signs but without weight loss, which is obviously different from that in the hMPV-infected mice [24]. This difference of clinical signs in the aMPV/C-inoculated mice may be related to virus specific as well as volume of virus inoculum (10^4.25^ TCID_50_ of aMPV/C in the present study vs 10^8.0^ TCID_50_ of hMPV in other study). However, the exact mechanism of clinical signs induced by aMPV/C inoculation needs to be further studied.

### Histopathological lesions in lungs of mice

**Figure 3:**
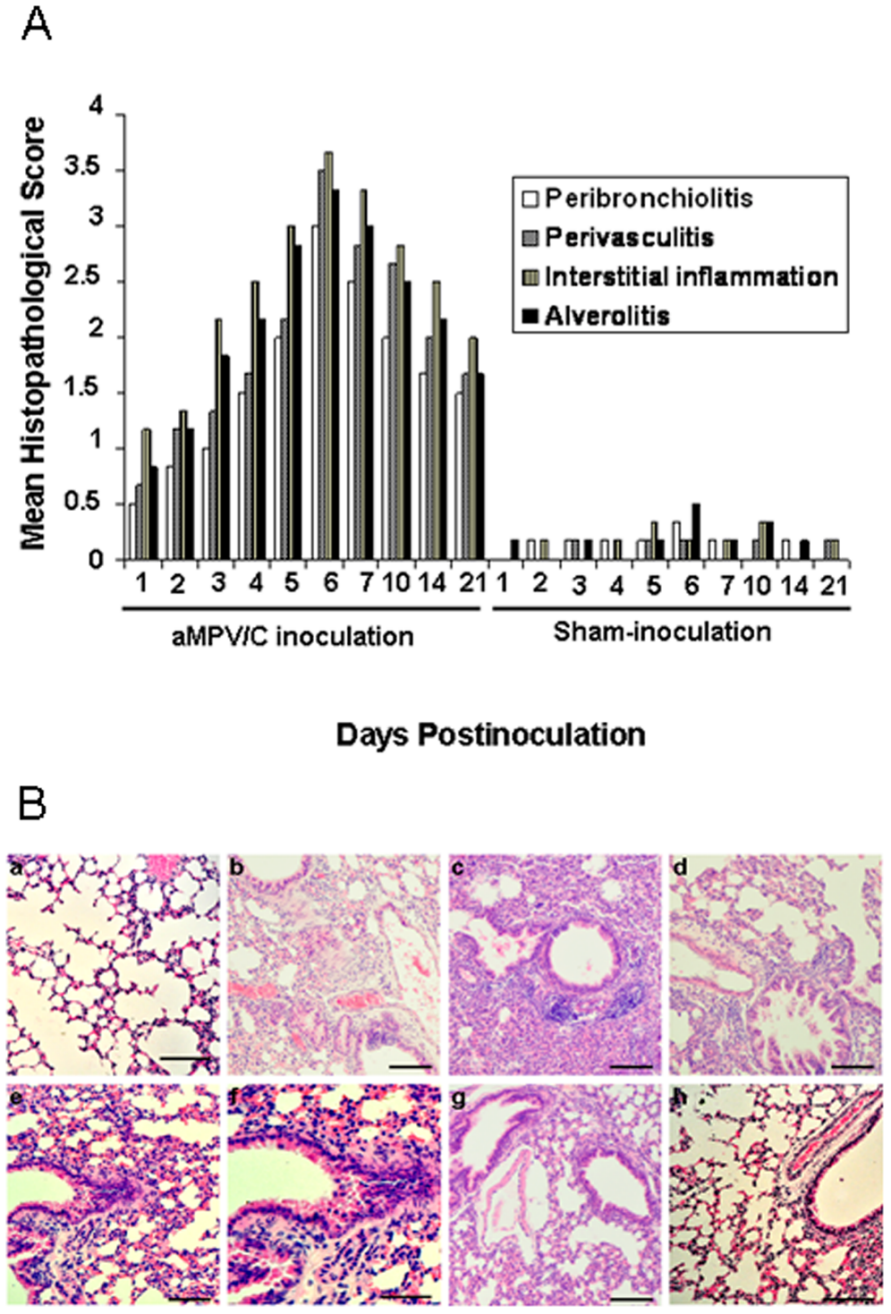
Histological appearance of the lungs of mice after aMPV/C inoculation. Six mice per group were sacrificed at the indicated times postinoculation, and their lung sections were stained with hematoxylin and eosin. (A) Histopathological scores in aMPV/C- and sham-inoculated mice. The mean lung histopathological score was evaluated based upon peribronchial, perivascular, interstitial, and alveolar areas. (B) (a) Normal morphology of lung section from a sham-inoculated mouse shows no significant inflammation. Representative lung sections are shown for the aMPV/C-inoculated mice on b (day 2), c (day 3), d (day 4), e (day 6), f (partial enlargement of panel e), g (day 10), and h (day 21) postinoculation. Lung lesions mainly consisted of interstitial edema and inflammatory cell infiltration near the small blood vessels and bronchioles, dropout of mucous epithelial cells in the bronchioles, thickening of the bronchial submucosa and alveolar walls, and alveolar lumen flooding with dropout of epithelial cells, erythrocytes, and inflammatory cells, such as lymphocytes, neutrophils, and plasmacytes. Bars, 80 μM.

The lung histopathology of the mice was examined at the indicated time points after aMPV/C inoculation. Pulmonary inflammation was assessed using a scoring scale system previously described [15], [24], [25]. As shown in Fig. 3A, the highest histopathological score occurred at the time of maximal virus replication (day 6) in the lungs of aMPV/C-inoculated mice. Lung lesions of the inoculated mice were characterized by interstitial edema and inflammatory cell infiltration near the small blood vessels and bronchioles, thickening of the bronchial submucosa and alveolar walls, and alveolar lumen flooding with dropout of epithelial cells, erythrocytes, and inflammatory cells, including mainly lymphocytes, neutrophils, and plasmacytes (Fig. 3B and data not shown). Sloughed epithelial cells, neutrophils, macrophages, and scant cellular debris and mucus were also visible in the bronchial lumens. Significant lesion was observed on days 2 to 10 postinoculation. At day 21 postinoculation, the lungs of the mice exhibited mild pathological lesions, such as mild inflammatory cell infiltration around the bronchioles and small blood vessels, and peribronchovascular congestion. No obvious histopathological changes were seen in sections of the lung in any sham-inoculated mice (Fig. 3A and B).

### aMPV antigen of lungs by immunohistochemical analysis

**Figure 4:**
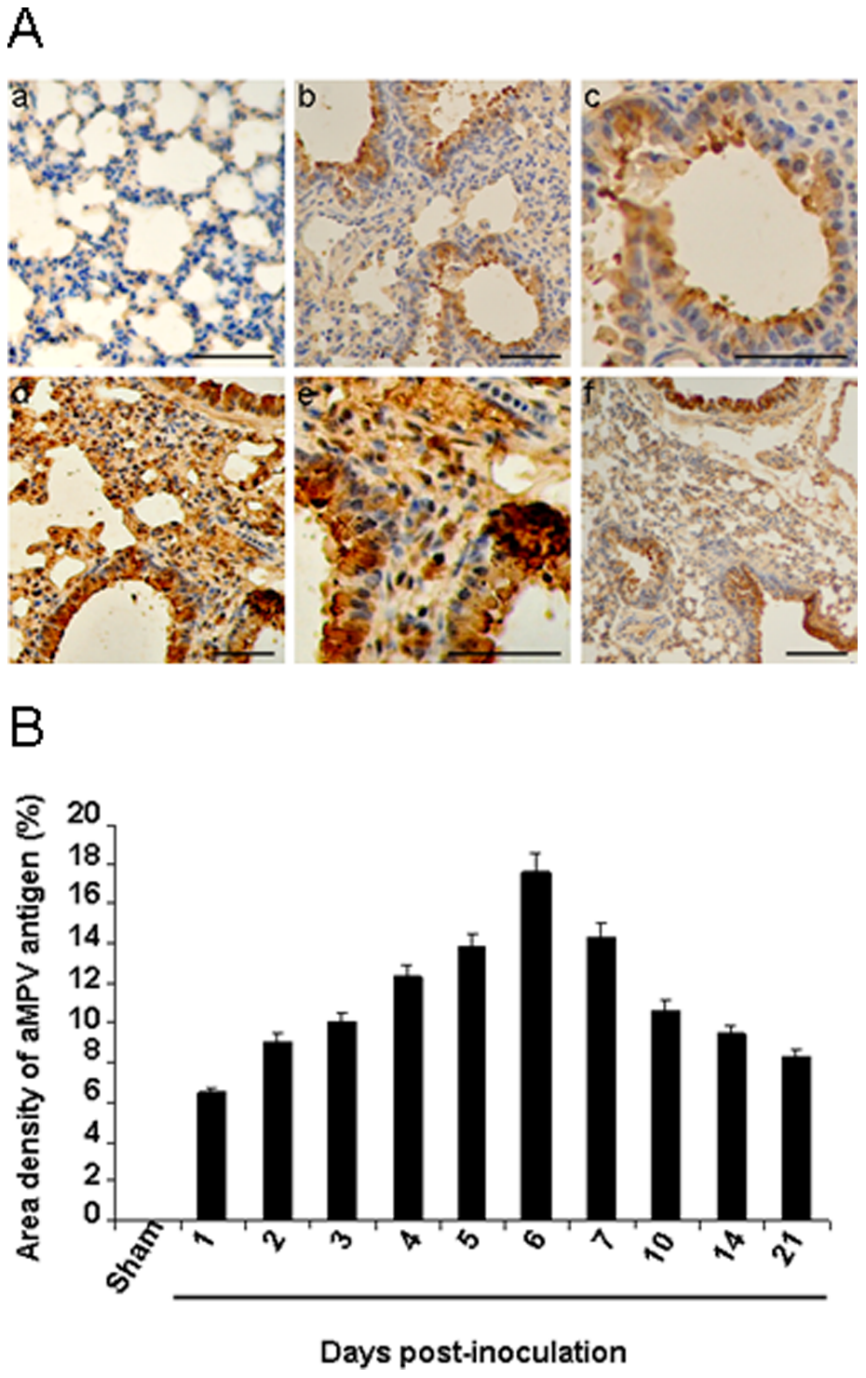
Immunohistochemical staining of the lungs of mice inoculated with aMPV/C strain JC. A. aMPV/C antigen-positive cells were brown. (a) No staining is observed in the lung section from a sham-inoculated mouse. (b) Lung tissue of the aMPV/C-inoculated mice at day 2 postinoculation shows many positive cells for aMPV/C antigen. (c) Partial enlargement of panel b. (d) Lung tissue of the aMPV/C-inoculated mice at day 6 postinoculation shows many positive cells for aMPV/C antigen. (e) Partial enlargement of panel d. (f) Lung tissue of the aMPV/C-inoculated mice at day 10 postinoculation shows positive cells for aMPV/C antigen. Bars, 80 μM. B. Dynamic changes of aMPV/C viral antigen in the lungs. Area density of aMPV/C positive signals in sham- and aMPV/C-inoculated mice at various time points postinoculation was showed. No aMPV/C viral antigens present in the sham-inoculated mice.

The lung tissues at the indicated times after aMPV/C inoculation were further subjected to immunohistochemical analysis for detecting the presence of aMPV viral antigen. As shown in Fig. 4A, lung tissue sections from the sham-inoculated mice did not exhibit reactivity with the antibody. For lung from the inoculated mice, aMPV/C antigen was detected extensively in intra-alveolar macrophages and pneumocytes, at the epithelial cells of the bronchioles. In addition, luminal cellular debris, including sloughed epithelial cells and macrophages, stained positive for aMPV/C antigen. To further determine aMPV-positive cells quantitatively in the inoculated mice, we determined an area density of positive signals. Consistent with the results shown in Fig. 2A and B, there was a time-dependent increase in the positive signals in the lungs of the aMPV/C-inoculated mice until 6 day postinoculation (with the maximal numbers as 17.65%) and then decreased (Fig. 4B). This is consistent with that virus titer and virus load in the lungs of mice (Fig. 2A and B). These data indicate that the chicken aMPV/C isolate investigated in the study can replicate efficiently in the lung of BALB/c mice thereby inducing histopathological changes, with the lesion severity being directly proportional to amounts of viral expression.

### Pulmonary cytokine/chemokine responses to aMPV/C inoculation

**Figure 5:**
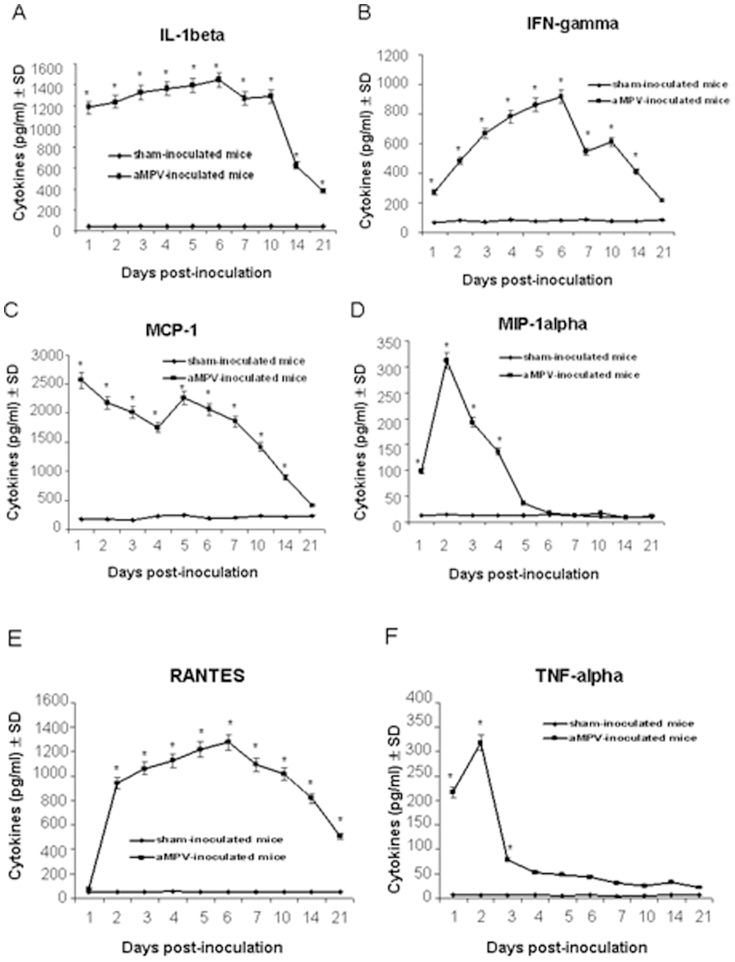
Cytokine/chemokine levels in lung homogenates of aMPV/C- and sham-inoculated mice. Six mice from both aMPV/C- and sham-inoculated groups were sacrificed on days 1, 2, 3, 4, 5, 6, 7, 10, 14, and 21 post-inoculation, and 100 μl of lung homogenates was used to quantify IL-1β (A), IFN-γ (B), MCP-1 (C), MIP-1α (D), RANTES (E), and TNF-α (F) by enzyme-linked immunosorbent assay. Significant difference is expressed as *p*<0.05 (*) for a comparison of aMPV/C-inoculated and sham-inoculated mice at the indicated time points after inoculation. Data are shown as the mean ± standard deviations (SD).

To further characterize the factors that regulate aMPV/C pathogenesis in the mouse model, we analyzed the production of cytokines/ckemokines in the lungs. As shown in Fig. 5, levels of IL-1β, IFN-γ, and RANTES peaked on day 6 and IL-1β continued to persist until day 21 post-inoculation in the lungs of aMPV/C-inoculated mice; such levels were significantly increased as compared to those in the sham-inoculated mice. MCP-1 was detected at the highest level on day 1 post-inoculation. MIP-1α and TNF-α peaked on day 2 thereafter declined. In contrast, no obvious IFN-α change was detected in the aMPV/C-inoculated mice as compared to the sham-inoculated mice throughout the experiment (data not shown).

### Neutralizing antibody

We further determined aMPV/C neutralizing antibody titers of pooled serum (*n*  =  6) collected from each time point after aMPV/C inoculation. Serum neutralizing antibody titers to aMPV/C were undetectable until day 14 postinoculation (with an antibody titer being 1:8) which increased thereafter. At day 21 postinoculation, the inoculated mice developed a statistically rise in aMPV/C-neutralizing serum titers (1:32), which are consistent with the significant decrease of aMPV/C amounts in the lungs from 14 to 21 day (Fig. 2A and B as well as Fig. 4B) after inoculation throughout the experiment. Regulation of virus replication may stimulate the production of serum aMPV/C-specific neutralizing antibody. However, the result also indicates that infectious aMPV/C persists in the lungs despite the presence of a neutralizing antibody as observed for hMPV infection in mice [16]. No detectable antibody was observed in the sham-inoculated mice.

## Discussion

Previous research showed that infection of turkeys with aMPV/C occurred primarily in the ciliated epithelial cells of the upper respiratory tract, exhibiting superficial erosive and inflammatory changes [27]. However, Cha et al. [28] recently demonstrated the presence of the viral genome in the turkey embryonic lung but without detectable pathological lesions after *in ovo* aMPV/C infection. In contrast to aMPV/C infection in turkeys, the results in the present study show that the chicken aMPV/C isolate which caused significant pathogenesis in chickens [20] can also lead to severe pathological inflammatory lesions in the lung of mice, which was characterized by infiltration of inflammatory cells, hyperplasia, interstitial edema, and thickening of the alveolar walls (Fig. 3B). In addition, inflammatory response characterized mainly by mild peribronchiolitis and perivasculitis was still present in the lungs of mice sacrificed on day 21, while infectious viruses could be detected and specific neutralizing antibody was produced. This may indicate a state of persistence in the aMPV/C-inoculated mice as observed for human respiratory syncytial virus (hRSV) infection of mice [29]. Such persistent inflammatory changes have also been described for more than 2 months in BALB/c mice following hMPV infection [23]. Alvarez and Tripp [16] reported that hMPV RNA can be detected in the lungs of BALB/c mice for up to 180 days in which impaired viral clearance could be the results of weak innate immune responses and aberrant adaptive immune responses after hMPV infection. In contrast to these findings, Darniot et al. [19] reported that the virus was cleared from the lungs of mice at day 9 after hMPV infection, regardless of aged or young mice. These conflicting results can be attributable possibly to the selection of the virus strain, thereby affecting virulence and pathogenesis.

An excessive and sustained inflammatory cytokine and chemokine production has been shown to be associated with the severity of viral diseases by causing severe local inflammation and tissue damage, such as hRSV and hMPV infections[19], [30], [31]. In the present study, high levels of varieties of inflammatory cytokines and chemokines such as TNF-α, IL-1β, IFN-γ, MCP-1, MIP-1α, and RANTES in the lungs of aMPV/C-inoculated mice (Fig. 5). As observed for hMPV infection in BALB/c mice [24], levels of the chemokines RANTES and the cytokine IFN-γ in the lungs of aMPV/C-inoculated peaked at the time of maximal viral replication (Fig. 4B and 5). However, the highest level of MIP-1α was not occurred at the time of the maximal viral replication but at day 2 post-inoculation (Fig. 4B and Fig. 5). This may be associated with enhanced inflammatory severity after aMPV/C infection, as observed for hRSV infection in the mouse model [32]. In accordance with that of Darniot et al. [19], the highest level of TNF-α expression was observed in the lung of aMPV/C-inoculated mice on day 2 post-inoculation (Fig. 5). High level of TNF-α involved in inflammation and fever may take responsible for clinical signs of illness such as fever that occurred during the few early days after aMPV/C inoculation (Fig. 1). IL-1β is a potent stimulator of neutrophil recruitment and is required for pathogen infections. Guerrero-Plata et al. [33] reported that hMPV poorly activated inflammatory cytokine IL-1β, which is in contrast to a recent report that IL-1β was significantly increased in the hMPV-infected mice [25]. In the present study, we observed that expression of IL-1β peaked at the time of maximal viral replication and persisted throughout the experiment (Fig. 5). These discrepancies can be due possibly to the different origins of the virus isolates as well as passage times and inocula amounts used for the mouse models. Together, these results indicated that varieties of inflammatory cytokine and chemokine productions occurred coordinately in the lungs of mice following aMPV/C inoculation, correlating with increased aMPV/C replication and/or severity of pulmonary inflammatory lesions thereby developing persistence of infection.

In conclusion, the results reported here indicate that the aMPV/C can replicate in the lungs of BALB/c mice with one growth kinetic in which peak viral amounts occurred at day 6 postinoculation. Of note, clinical signs of illness consisted of fever and some breathing difficulties that occurred on days 1 to 5 postinoculation. Pathological changes were characterized by an increased number of alvaeolar macrophages as well as infiltrated inflammatory cells including mainly lymphocytes, neutrophils, and plasmacytes in the lungs of aMPV/C-inoculated mice. The close relatedness between the lung lesions, the presence of aMPV/C antigen, and high levels of pulmonary inflammatory cytokines and chemokines including MCP-1, MIP-1α, RANTES, IL-1β, IFN-γ, and TNF-α, together with clinical signs in the aMPV/C-inoculated mice and the absence of these changes in the sham-inoculated control tissues, support our conclusion that chicken aMPV/C can infect BALB/c mice and induce persistent infection. We demonstrated here that chicken aMPV/C behaviours similar to hMPV in mice. However, whether this chicken aMPV/C has potential as zoonotic pathogen, further investigation will be required.

## References

[1] McDougall JS, Cook JKA (1986) Turkey rhinotracheitis: preliminary investigations. Vet Rec 118: 206–207. 3716162 10.1136/vr.118.8.206

[2] Easton AJ, Domachowske JB, Rosenberg HF (2004) Animal pneumoviruses: molecular genetics and pathogenesis. Clin Microbiol Rev 17: 390–412. 15084507 10.1128/CMR.17.2.390-412.2004 PMC387412

[3] Buys SB, Du Preez JH (1980) A preliminary report on the isolation of a virus causing sinusitis in turkeys in South African and attempts attenuate the virus. Turkeys 28: 36–46.

[4] Cook JKA, Huggins MB, Orbell SJ, Senne DA (1999) Preliminary antigenic characterization of an avian Pneumovirus isolated from a commercial turkeys in Colorado, USA. Avian Pathol 28: 607–617. 10.1080/03079459994407 27266432

[5] Toquin D, Guionie O, Jestin V, Zwingelstein F, Allee C, et al (2006) European and American subgroup C isolates of avian metapneumovirus belong to different genetic lineages. Virus Gen 32: 97–103. 10.1007/s11262-005-5850-3 16525740

[6] Lee E, Song MS, Shin JY, Lee YM, Kim CJ, et al (2007) Genetic characterization of avian metapneumovirus subtype C isolated from pheasants in a live bird market. Virus Res 128: 18–25. 17485129 10.1016/j.virusres.2007.03.029

[7] Turpin EA, Stallknecht DE, Slemons RD, Zsak L, Swayne DE (2008) Evidence of avian metapneumovirus subtype C infection of wild birds in Georgia, South Carolina, Arkansas and Ohio, USA. Avian Pathol 37: 343–351. 18568663 10.1080/03079450802068566

[8] Nijenga MK, Lwamba HM, Seal BS (2003) Metapneumoviruses in birds and humans. Virus Res 91: 163–169. 12573494 10.1016/s0168-1702(02)00256-3

[9] van den Hoogen BG, Bestebroer BG, Osterhaus AD, Fouchier RA (2002) Analysis of the genomic sequence of a human metapneumovirus. Virology 295: 119–132. 12033771 10.1006/viro.2001.1355

[10] Yunus AS, Govindarajan D, Huang Z, Samal SK (2003) Deduced amino acid sequence of the small hydrophobic protein of US avian pneumovirus has greater identity with that of human metapneumovirus than those of non-US avian pneumoviruses. Virus Res 93: 91–97. 12727346 10.1016/s0168-1702(03)00074-1

[11] Boivin G, Abed Y, Pelletier G, Ruel L, Moisan D, et al (2002) Virological features and clinical manifestations associated with human metapneumovirus: a new paramyxovirus responsible for acute respiratory-tract infections in all age groups. J Infect Dis 186: 1330–1334. 12402203 10.1086/344319

[12] Kuiken T, van den Hoogen BG, van Riel DA, Laman JD, Van Amerongen G, et al (2004) Experimental human metapneumovirus infection of cynomolgus macaques (Macaca fascicularis) results in virus replication in ciliated epithelial cells and pneumocytes with associated lesions throughout the respiratory tract. Am J Pathol 164: 1893–1900. 15161626 10.1016/S0002-9440(10)63750-9 PMC1615765

[13] MacPhail M, Schickli JH, Tang RS, Kaur J, Robinson C, et al (2004) Identification of small-animal and primate models for evaluation of vaccine candidates for human metapneumovirus (hMPV) and implications for hMPV vaccine design. J Gen Virol 85: 1655–1663. 15166450 10.1099/vir.0.79805-0

[14] Skiadopoulos MH, Biacchesi S, Buchholz UJ, Riggs JM, Surman SR, et al (2004) The two major human metapneumovirus genetic lineages are highly related antigenically, and the fusion (F) protein is a major contributor to this antigenic relatedness. J Virol 78: 6927–6937. 15194769 10.1128/JVI.78.13.6927-6937.2004 PMC421687

[15] Alvarez R, Harrod KS, Shieh WJ, Zaki S, Tripp RA (2004) Human metapneumovirus persists in BALB/c mice despite the presence of neutralizing antibodies. J Virol 78: 14003–14011. 15564507 10.1128/JVI.78.24.14003-14011.2004 PMC533920

[16] Alvarez R, Tripp RA (2005) The immune response to human metapneumovirus is associated with aberrant immunity and impaired virus clearance in BALB/c mice. J Virol 79: 5971–5978. 15857983 10.1128/JVI.79.10.5971-5978.2005 PMC1091678

[17] Chakraborty K, Zhou Z, Wakamatsu N, Guerrero-Plata A (2012) Interleukin-12p40 modulates human metapneumovirus-induced pulmonary disease in an acute mouse model of infection. PLoS ONE 7(5), e37173. doi:10.1371/journal.pone.0037173 10.1371/journal.pone.0037173 PMC3351396 22606349

[18] Darniot M, Petrella T, Aho S, Pothier P, Manoha C (2005) Immune response and alteration of pulmonary function after primary human metapneumovirus (hMPV) infection of BAL/c mice. Vaccine 23: 4473–4480. 15927322 10.1016/j.vaccine.2005.04.027

[19] Darniot M, Pitoiset C, Petrella T, Aho S, Pothier P, et al (2009) Age-associated aggravation of clinical disease after primary metapneumovirus infection of BALB/c mice. J Virol 83: 3323–3332. 19144706 10.1128/JVI.02198-08 PMC2655584

[20] Wei L, Zhu S, Yan X, Wang J, Zhang C, et al (2013) Avian metapneumovirus subgroup C infection in chickens, China. Emerg Infect Dis 19: 1092–1094. 23763901 10.3201/eid1907.121126 PMC3903454

[21] Velayudhan BT, Nagaraja KV, Thachil AJ, Shaw DP, Gray GC, et al (2006) Human metapneumovirus in turkey poults. Emerg Infect Dis 12: 1853–1859. 17235379 10.3201/eid1212.060450 PMC1776506

[22] World Organization for Animal Health (2011) Terrestrial animal health code. Paris: World Organization for Animal Health.

[23] Hamelin ME, Prince GA, Gomez AM, Kinkead R, Boivin G (2006) Human metapneumovirus infection induces long-term pulmonary inflammation associated with airway obstruction and hyperresponsiveness in mice. J Infect Dis 193: 1634–1642. 16703506 10.1086/504262

[24] Hamelin ME, Yim K, Kuhn KH, Cragin RP, Boukhvalova M, et al (2005) Pathogenesis of human metapneumovirus lung infection in BALB/c mice and cotton rats. J Virol 79: 8894–8903. 15994783 10.1128/JVI.79.14.8894-8903.2005 PMC1168778

[25] Kukavica-Ibrulj I, Hamelin ME, Prince GA, Gagnon C, Bergeron Y, et al (2009) Infection with human metapneumovirus predisposes mice to severe pneumococcal pneumonia. J Virol 83: 1341–1349. 19019962 10.1128/JVI.01123-08 PMC2620891

[26] Alvarez R, Njenga MK, Scott M, Seal BS (2004) Development of a nucleoprotein-based enzyme-linked immunosorbent assay using a synthetic peptide antigen for detection of avian metapneumovirus antibodies in turkey sera. Clin Diagn Lab Immun 11: 245–249. 10.1128/CDLI.11.2.245-249.2004 PMC371206 15013970

[27] Jirjis FF, Noll SL, Halvorson DA, Nagaraja KV, Shaw DP (2002) Pathogenesis of avian pneumovirus infection in turkeys. Vet Pathol 39: 300–310. 12014494 10.1354/vp.39-3-300

[28] Cha RM, Khatri M, Mutnal M, Sharma JM (2011) Pathogenic and immunogenic responses in turkeys following in ovo exposure to avian metapneumovirus subtype C. Vet Immunol Immunopathol. 140: 30–36. 10.1016/j.vetimm.2010.11.006 21146877

[29] Schwarze J, O’Donnell DR, Rohwedder A, Openshaw PJ (2004) Latency and persistence of respiratory syncytial virus despite T cell immunity. Am J Respir Crit Care Med 169: 801–805. 14742302 10.1164/rccm.200308-1203OC

[30] Durbin JE, Johnson TR, Durbin RK, Mertz SE, Morotti RA, et al (2002) The role of IFN in respiratory syncytial virus pathogenesis. J Immunol 168: 2944–2952. 11884466 10.4049/jimmunol.168.6.2944

[31] Haeberle HA, Kuziel WA, Dieterich HJ, Casola A, Gatalica Z, et al (2001) Inducible expression of inflammatory chemokines in respiratory syncytial virus-infected mice: role of MIP-1α in lung pathology. J Virol 75: 878–890. 11134301 10.1128/JVI.75.2.878-890.2001 PMC113984

[32] Jafri HS, Chavez-Bueno S, Mejias A, Gomez AM, Rios AM, et al.. (2004) Respiratory syncytial virus induces pneumonia, cytokine response, airway obstruction, and chronic inflammatory infiltrates associated with long-term airway hyperresponsiveness in mice. J Infect Dis 189, 1856–1865. 10.1086/386372 15122522

[33] Guerrero-Plata A, Casola A, Garofalo RP (2005) Human metapneumovirus induces a profile of lung cytokines distinct from that of respiratory syncytial virus. J Virol 79: 14992–14997. 16282501 10.1128/JVI.79.23.14992-14997.2005 PMC1287587

